# ﻿Unveiling species diversity within the family Conidiobolaceae (Entomophthorales) in China: Descriptions of two new species and reassessment of the taxonomic position of *Conidioboluspolyspermus*

**DOI:** 10.3897/mycokeys.105.117871

**Published:** 2024-05-22

**Authors:** Yong Nie, Ying Yin, Heng Zhao, XiaoYong Liu, Bo Huang

**Affiliations:** 1 Anhui Provincial Key Laboratory for Microbial Pest Control, Anhui Agricultural University, Hefei 230036, China Anhui University of Technology Hefei China; 2 School of Civil Engineering and Architecture, Anhui University of Technology, Ma,anshan 243002, China Anhui Agricultural University Hefei China; 3 Institute of Microbiology, School of Ecology and Nature Conservation, Beijing Forestry University, Beijing 100083, China Beijing Forestry University Beijing China; 4 College of Life Sciences, Shandong Normal University, Jinan 250014, China Shandong Normal University Jinan China

**Keywords:** Basal fungi, *EFL*, mtSSU, new species, nucLSU, taxonomic position

## Abstract

In the present study, two new *Conidiobolus* s.s. species were described relying on the morphological studies and phylogenetic analysis utilizing nuclear large subunit of rDNA (nucLSU), mitochondrial small subunit of rDNA (mtSSU), and elongation-factor-like gene (*EFL*) sequences. *Conidiobolusjiangxiensis***sp. nov.** is distinguished by its short primary conidiophores, a feature not commonly observed in other *Conidiobolus* s.s. species. Conversely, *Conidiobolusmarcoconidius***sp. nov.** is characterized by larger primary conidia and the emergence of 2–5 secondary conidia from each branched secondary conidiophores. Additionally, the taxonomic reassessment of *C.polyspermus* confirms its distinct status within the genus *Conidiobolus* s.s. Moreover, molecular analyses, incorporating the nucLSU, mtSSU, and *EFL* sequences, provide robust support for the phylogenetic placement of the two newly described species and the taxonomic identity of *C.polyspermus*. This investigation contributes valuable insights into the species diversity of *Conidiobolaceae* in China, enhancing our understanding of the taxonomy within this fungal family.

## ﻿Introduction

The reclassification of conidiobolus-like fungi into three families based on phylogenetic and morphological evidence led to the establishment of the family Conidiobolaceae (Entomophthorales), housing three genera i.e. *Azygosporus* B. Huang & Y. Nie, *Conidiobolus* s.s. B. Huang & Y. Nie, and *Microconidiobolus* B. Huang & Y. Nie (Gryganskyi et al. 2022).Members of this family primarily exhibit a saprobic lifestyle, thriving in soil and plant debris. However, exceptions exist, with *C.coronatus* distinguished for its capacity to infect both insects and humans, and *C.lunulus* being isolated from leafcutter ants in Argentina (Goffre et al. 2020; Möckel et al. 2022).

A comprehensive taxonomy of conidiobolus-like fungi has identified *Conidiobolus* s.s. as distinct from four other genera (Nie et al. 2020a, b, 2023; Cai et al. 2021). However, the synonymy of *C.megalotocus* (= *C.polyspermus*, = *C.eurypus*), introduced by King (1976b), remains unexplored, and requiring re-evaluation. Additionally, our previous study confirmed the synonymy of *C.firmipilleus* (= *C.chlamydosporus*). Thus, this study aims to add taxonomic clarity by re-evaluating the aforementioned synonym and introducing additional taxonomic taxa.

Previous phylogenetic analyses revealed two main clades within *Conidiobolus* s.s. when Capillidiaceae and Neoconidiobolaceae species were used as outgroups (Nie et al. 2020a, b; Gryganskyi et al. 2022). However, distinctive traits within each clade were not elucidated. Notably, both clades included species lacking microspore observations, such as *C.iuxtagenitus*, *C.margaritatus*, *C.lichenicolus*, *C.taihushanensis*, *C.dabieshanensis*, and *C.longiconidiophorus* (Srinivasan and Thirumalachar 1968; Waters and Callaghan 1989; Huang et al. 2007; Nie et al. 2017, 2020b, 2023). Particularly, *C.iuxtagenitus*, potentially forming a separate lineage, produces fusiform secondary conidia, and its zygospores form via a short beak near lateral conjugation (Waters and Callaghan 1989; Nie et al. 2023). Regrettably, molecular data for *C.margaritatus* are currently unavailable, leaving unanswered questions about the relationships among these morphologically distinct species and the possibility of undiscovered lineages within this fungal group. Resolving these issues requires additional members for phylogenetic and morphological studies.

Over the last decades, only six new *Conidiobolus* s.s. species and three new records were reported from China (Wang et al. 2010; Nie et al. 2017, 2020b, 2023). Additionally, the understanding of phylogenetic relationships within the accepted species of *Azygosporus* and *Microconidiobolus* remains limited. Consequently, a comprehensive exploration of the species diversity within *Conidiobolaceae* is imperative to unravel the relationships within this intricate fungal group. This article aims to identify two new *Conidiobolus* s.s. species using morphological characters and phylogenetic analyses of nucLSU, mtSSU, and *EFL* sequences. Simultaneously, the taxonomic status of *C.polyspermus* will be clarified.

## ﻿Materials and methods

### ﻿Isolation and morphology

Plant debris and soil samples were collected from Dashushan and Binhu National Forest Park, Hefei City, Anhui Province, and Aixihu Forest Wetland Park, Nanchang City, Jiangxi Province, during 2022. For isolation of conidiobolus-like fungi, we are following the previous described methods (King 1976a; Nie et al. 2012). All samples were preserved in sterilized plastic bags and transported to the laboratory as soon as possible. Plant debris samples were cut into several approximately 2 cm sized fragments and placed evenly on the Petri dishes cover. Then, using a Petri dish with potato dextrose agar (PDA; potato 200 g, dextrose 20 g, agar 20 g, H_2_O 1 L) inverted over the treated samples to obtain discharged conidia, and incubating at 21 °C for daily examining by a stereomicroscope (SMZ1500, Nikon Corporation, Japan) for 7 days. When conidiobolus-like fungi observed, they were transferred to new PDA plate for purification and morphological observation.

The micro-morphological structure of mycelium, primary conidia and conidiophores, secondary conidia, and resting spores at 400× magnification was observed under a BX51 microscope (Olympus Corporation, Tokyo, Japan) and imaged using a DP25 microscope-camera system (Olympus Corporation, Tokyo, Japan) under differential interference contrast (DIC) condition. Each character was made more than 35 measurements and the description was made with the method by King (1976a). The purification isolates were deposited at the Engineering Research Center of Biofilm Water Purification and Utilization Technology of Ministry of Education at Anhui University of Technology, Anhui Province, China (BWPU), and duplicated at Research Center for Entomogenous Fungi at Anhui Agricultural University, Anhui Province, China (RCEF). In addition, 14 ex-types of *Conidiobolus* s.l. were purchased from the American Type Culture Collection, Manassas, VA, USA (ATCC).

### ﻿DNA extraction, PCR amplification and sequencing

Pure cultures were grown on PDA for 7 days at 21 °C. Fresh fungal mycelia were scraped from the surface of PDA and transferred to Eppendorf tubes. Genomic DNA was extracted using a modified cetyltrimethylammonium bromide (CTAB) method (Watanabe et al. 2010). Primers used for PCR amplification of the nucLSU (LR0R/LR5), mtSSU (mtSSU1/mtSSU2R), and *EFL* (EF983/EF1aZ-1R) genes were followed as described previously (Vilgalys and Hester 1990; Zoller et al. 1999; Nie et al. 2012).

DNA amplification was performed in a 50 μl reaction volume which contained 1 μL dNTPs (200 μM), 1 μL MgCl_2_ (2.5 mM), 10 µL Phusion HF buffer (5×), 1 μL primers each (0.5 μM), 100 ng genomic DNA, and 0.5 μL Taq polymerase (0.04 Unit/L, Super Pfx DNA Polymerase, Cowinbioscience Co. Ltd., Shanghai, China). PCR amplificated program followed Nie et al. (2020a). Sequencing was generated by Shanghai Genecore Biotechnologies Company (Shanghai, China), and were processed with Geneious 9.0.2 (Kearse et al. 2012) to obtain consensus sequences. All sequences were deposited in GenBank (Table 1).

**Table 1.** The species useduin phylogenetic analyses.

Species	Strains*	GenBank accession numbers		
		nucLSU	*EFL*	mtSSU
*Azygosporusmacropapillatus*	CGMCC 3.16068 (T)	MZ542006	MZ555650	MZ542279
*parvus*	ATCC 14634 (T)	KX752051	KY402207	MK301192
*Conidiobolusbifurcatus*	CGMCC 3.15889 (T)	MN061285	MN061482	MN061288
*C.brefeldianus*	ARSEF 452 (T)	EF392382	–	EF392495
*C.chlamydosporus*	ATCC 12242 (T)	JF816212	JF816234	MK301178
*C.coronatus*	NRRL 28638	AY546691	DQ275337	–
*C.coronatus*	RCEF 4518	JN131537	JN131543	–
*C.dabieshanensis*	CGMCC 3.15763 (T)	KY398125	KY402206	MK301180
*C.firmipilleus*	ARSEF 6384	JX242592	–	JX242632
*C.gonimodes*	ATCC 14445 (T)	JF816221	JF816226	MK301182
*C.humicolus*	ATCC 28849 (T)	JF816220	JF816231	MK301184
*C.incongruus*	NRRL 28636 (T)	AF113457	–	–
*C.iuxtagenitus*	ARSEF 6378 (T)	KC788410	–	–
*C.iuxtagenitus*	RCEF 4445	JX946695	JX946700	MK333391
***C.jiangxiensis* sp.nov.**	**RCEF 7484 (T)**	**PP034291**	**PP035215**	**PP034295**
***C.jiangxiensis* sp.nov.**	**RCEF 7485**	**PP034292**	**PP035216**	**PP034296**
*C.khandalensis*	ATCC 15162 (T)	KX686994	KY402204	MK301185
*C.lichenicolus*	ATCC 16200 (T)	JF816216	JF816232	MK301186
*C.longiconidiophorus*	RCEF 6563 (T)	OQ540746	OQ550509	OQ540744
***C.marcoconidius* sp.nov.**	**RCEF 6918 (T)**	**PP034289**	**PP035213**	**PP034293**
***C.marcoconidius* sp.nov.**	**RCEF 7412**	**PP034290**	**PP035214**	**PP034294**
*C.marcosporus*	ATCC 16578 (T)	KY398124	KY402209	MK301188
*C.megalotocus*	ATCC 28854 (T)	MF616383	MF616385	MK301189
*C.mycophagus*	ATCC 16201 (T)	JX946694	JX946698	MK301190
*C.mycophilus*	ATCC 16199 (T)	KX686995	KY402205	MK301191
*C.polyspermus*	ATCC 14444 (T)	MF616382	MF616384	MK301193
*C.polysporus*	RCEF 7058 (T)	OQ540747	OQ550510	OQ540745
*C.polytocus*	ATCC 12244 (T)	JF816213	JF816227	MK301194
*C.taihushanensis*	CGMCC 3.15900 (T)	MT250086	MT274290	MT250088
*C.variabilis*	CGMCC 3.15901 (T)	MT250085	MT274289	MT250087
*Microconidiobolusnodosus*	ATCC 16577 (T)	JF816217	JF816235	MK333388
*M.paulus*	ARSEF 450 (T)	KC788409	–	–
*M.terrestris*	ATCC 16198 (T)	KX752050	KY402208	MK301199

*ARSEF, ARS Entomopathogenic Fungus Collection (Ithaca, U.S.A.). ATCC, American Type Culture Collection (Manassas, U.S.A). CGMCC, China General Microbiological Culture Collection Center (Beijing, China). NRRL, ARS Culture Collection (Peoria, U.S.A). RCEF, Research Center for Entomogenous Fungi (Hefei, China). T = ex-type. The new species reported in this study are indicated in bold.

### ﻿Phylogenetic analyses

DNA sequences of three loci (nucLSU, mtSSU, and *EFL*) originated from *Conidiobolus* s.s. species were downloaded from GenBank database (Table 1) with two *Azygosporus* and three *Microconidiobolus* species served as outgroups. Sequence alignment of each locus was performed with MUSCLE 3.8.31 (Edgar 2004). Then, the alignments were checked and manually adjusted in BioEdit 7.0.1 (Hall 1999). The concatenated matrices were assembled by SequenceMatrix 1.7.8 (Vaidya et al. 2011). The obtained matrix was deposited in TreeBase (https://treebase.org) with the submission ID 31051. The best-fit likelihood models were estimated for each partition with MrModeltest v.2.3 (Nylander 2004). Maximum likelihood (ML) analyses were conducted with RAxML 8.1.17 using 1000 bootstrap replicates (Stamatakis 2014). Bayesian Inference (BI) analyses were calculated with MrBayes 3.2 (Ronquist and Huelsenbeck 2003). Bayesian posterior probabilities (PP) were estimated by the Metropolis-coupled Markov chain Monte Carlo method (Geyer 1991). Four simultaneous Markov chains were run starting from random trees for 1 million generations, keeping one tree every 100^th^ generation until the average standard deviation of split frequencies was below 0.01. The value of burn-in was set to discard 25% of trees and posterior probabilities (PP) were determined from the remaining trees. The phylogenetic tree was visualized in FigTree 1.4 (Rambaut 2012) and improved with Adobe Illustrator CS6.0.

## ﻿Results

### ﻿Phylogenetic analyses

**Figure 1.**
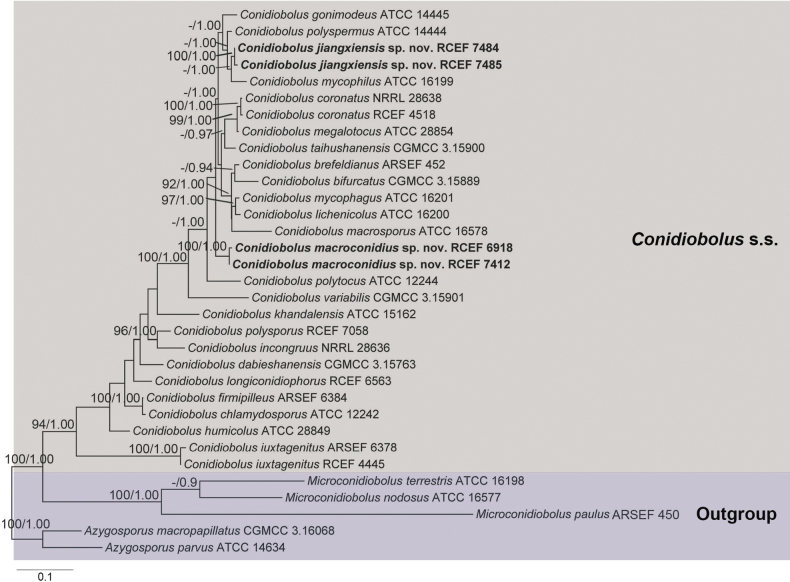
Maximum likelihood (ML) tree obtained by phylogenetic analyses of the combined nucLSU, *EFL* and mtSSU sequences. Two *Azygosporus* and three *Microconidiobolus* species were served as outgroups. The proposed new species is in boldface. Maximum Likelihood bootstrap values (≥70%) / Bayesian posterior probabilities (≥0.95) of clades are provided alongside the branches. The scale bar at the bottom left indicates substitutions per site.

The concatenated dataset comprised 1883 nucleotide sites, with specific contributions of 981 for nucLSU, 501 for SSU, and 401 for *EFL*. Within this dataset, 964 characters remained constant, 656 were parsimony-informative, and 308 were parsimony-uninformative. Model selection for individual data from each partition in both ML and BI phylogenetic analyses resulted in the application of the GTR+I+G model. The ML optimization likelihood reached a final value of -13813.01, and the average standard deviation of the split frequencies at the end of the analyses was 0.00619. The resulting phylogram from the ML analysis is depicted in Fig. 1.

Contrary to previous studies (Nie et al. 2020a, b; Gryganskyi et al. 2022), the current phylogenetic tree did not exhibit the grouping of all *Conidiobolus* s.s. members into two main clades. Notably, the phylogeny revealed that the four newly isolated strains (RCEF 6918, RCEF 7412, RCEF 7484, and RCEF 7485) were situated within the genus *Conidiobolus* s.s. Specifically, strains RCEF 7484 and RCEF 7485 clustered with *C.mycophilus*, garnering high to full support (100/1.00), while strains RCEF 6918 and RCEF 7412 formed a distinct clade with full support (100/1.00). Additionally, a subclade was formed by *C.polyspermus*, *C.mycophilus*, *C.gonimodes*, RCEF 7484, and RCEF 7485. However, *C.polyspermus* exhibited a distinct genetic distance from the other three species.

### ﻿Taxonomy

#### ﻿ Conidiobolus jiangxiensis

Taxon classificationFungiEntomophthoralesConidiobolaceae

B. Huang & Y. Nie sp. nov.

F3F83DFD-3AE9-5E02-8226-80D0FB03CF71

MycoBank No: 851495

Fig. 2

##### Etymology.

*jiangxiensis* (Lat.), referring to the region where the fungus was isolated.

##### Known distribution.

Jiangxi Province, China.

##### Typification.

China, Jiangxi Province, Nanchang City, Aixihu Forest Wetland Park, 28°69′N, 115°99′E, from soil, 7 Dec. 2022, *Y. Nie*, holotype BWPU 221207. Ex-type culture RCEF 7484. GenBank: nucLSU = PP034291; *EFL* = PP035215; mtSSU = PP034295.

**Figure 2.**
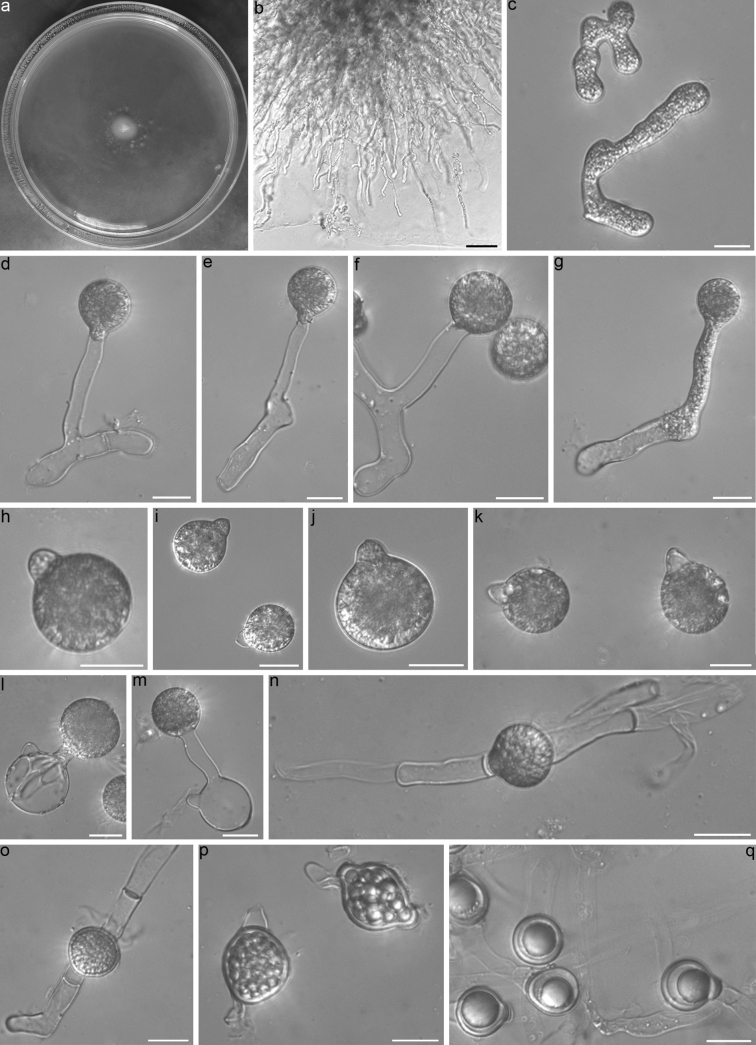
*Conidiobolusjiangxiensis*RCEF 7484 **a** colony on PDA after 3 d at 21 °C **b** mycelia unbranched at the edge of the colony **c** hyphal segments **d–g** primary conidiophores bearing a single primary conidia **h–k** primary conidia **l, m** primary conidia bearing a single secondary conidium **n, o** zygospores formed between adjacent segments of the same hypha **p** young zygospores **q** mature zygospores. Scale bars: 100 μm (**b**); 20 μm (**d–q**).

##### Additional specimens examined.

China, Jiangxi Province, Nanchang City, Aixihu Forest Wetland Park, 28°69′N, 115°99′E, from soil, 7 Dec. 2022, *Y. Nie* culture RCEF 7485. GenBank: nucLSU = PP034292; *EFL* = PP035216; mtSSU = PP034296.

##### Description.

Colonies on PDA at 21 °C after 3 d, white, reaching ca 11 mm in diameter. Mycelia white, 8–15 μm wide, often unbranched at the edge of colony, non-septate when young, and distended to segment after 7 d. Primary conidiophores often arising from hyphae, short, 30–95 × 7–10 μm, unbranched and producing a single primary conidium, without widening upward near the tip. Primary conidia forcibly discharged, globose to subglobose, 30–41 × 24–36 μm, papilla bluntly-round, 8–13 μm wide, 3.5–9 μm long. Secondary conidiophores arising from primary conidia, bearing a single similar but smaller replicative conidium to primary conidia. Microspores not observed on the PDA culture and on the 2% water agar. Zygospores formed in axial alignment with conjugating segments after 10 days, mature zygospores smooth, usually globose, sometimes subglobose, 20–30 μm in diameter, with a 2–3 μm thick wall.

##### Notes.

*Conidiobolusjiangxiensis*, *C.polyspermus* and *C.mycophilus* exhibit close phylogenetic relatedness. However, the primary conidia and zygospores of *C.jiangxiensis* are smaller than those of *C.polyspermus*, and *C.jiangxiensis* is further set apart from *C.mycophilus* by its longer primary conidiophores and larger primary conidia (Drechsler 1961; Srinivasan and Thirumalachar 1965). Despite the high similarities in nucLSU and *EFL* between *C.jiangxiensis* and *C.polyspermus*, their differentiation becomes evident through morphological traits. Similar instances of this phenomenon are observed in *C.coronatus* and *C.megalotocus*, as well as *C.mycophagus* and *C.lichenicolus*. Morphologically, *C.jiangxiensis* presents shorter primary conidiophores (no more than 95 μm) compared to the majority of other *Conidiobolus* s.s. members. It closely resembles *C.marcosporus* (50–100 μm), *C.lichenicolus* (30–100 μm), and *C.gonimodes* (20–80 μm) according to the length of primary conidiophores. Distinguishing features include its smaller primary conidia and zygospores in comparison to *C.marcosporus* (Srinivasan & Thirumalachar, 1967) and larger primary conidia, as well as the absence of primary conidia arising as upward branches from hyphal knots, distinguishing it from *C.lichenicolus* (Srinivasan and Thirumalachar 1968). Notably, *C.jiangxiensis* aligns with *C.gonimodes* based on primary conidia size, yet it differs by the distinct width of mycelia and the presence of unbranched primary conidiophores (Drechsler 1961). Furthermore, in the phylogenetic tree, *C.jiangxiensis* is distantly related to *C.gonimodes*. (Fig. 1)

#### ﻿ Conidiobolus marcoconidius

Taxon classificationFungiEntomophthoralesConidiobolaceae

B. Huang & Y. Nie sp. nov.

31A61AD9-4859-5A3F-8423-3D077AA0EA05

MycoBank No: 851496

Fig. 3

##### Etymology.

*marcoconidius* (Lat.), referring to its large primary conidia.

##### Known distribution.

Anhui Provinces, China.

##### Typification.

China, Anhui Province, Hefei City, Dashushan National Forest Park, 31°84′N, 117°17′E, from plant debris, 15 Mar. 2022, *Y. Yin*, holotype DSS 20220315. Ex-type culture RCEF 6918. GenBank: nucLSU = PP034289; *EFL* = PP035213; mtSSU = PP034293.

**Figure 3.**
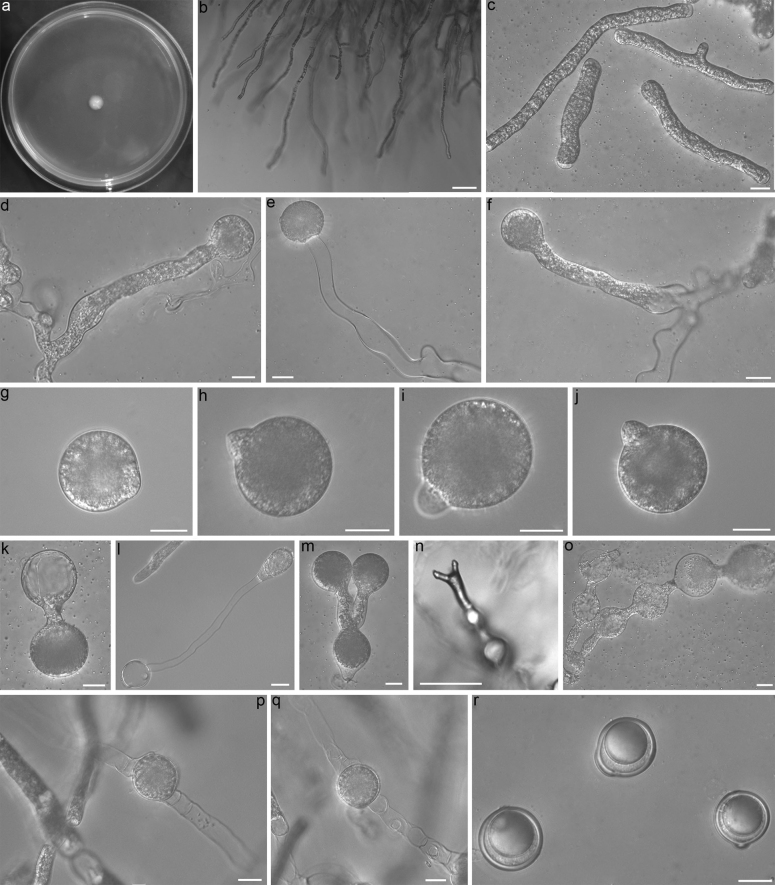
*Conidiobolusmarcoconidius*RCEF 6918 **a** colony on PDA after 3 d at 21 °C **b** mycelia unbranched at the edge of the colony **c** hyphal segments **d–f** primary conidiophores **g–j** primary conidia **k, l** primary conidia bearing a single secondary conidium **m** secondary conidiophore branched at the base and bearing two secondary conidia at each tip **n** secondary conidiophore branched at the tip **o** secondary conidiophore branched at the base bearing 2-5 secondary conidia at each branch **p, q** zygospores formed between adjacent segments of the same hypha **r** mature zygospores. Scale bars: 100 μm (**b, n**); 20 μm (**c–m, o–r**).

##### Additional specimens examined.

China, Anhui Province, Hefei City, Binhu National Forest Park, 31°73'N, 117°38'E, from plant debris, 10 May 2022, *Y. Yin*,, culture RCEF 7412. GenBank: GenBank: nucLSU = PP034290; *EFL* = PP035214; mtSSU = PP034294.

##### Description.

Colonies on PDA at 21 °C after 3 d white, reaching ca 8 mm in diameter. Mycelia colorless, unbranched at the edge of colony, distended to a width of 9–20 μm segment after 5 d. Primary conidiophores unbranched, slightly curved at the tip, producing a single primary conidium, without widening upward near the tip, 105–230 × 10–16 μm. Primary conidia forcibly discharged, mostly globose, sometimes obovoid, 45–67 × 42–58 μm, with a sharp or round papilla, 13–22 μm wide, 4–13 μm long. Secondary conidia arising from primary conidia, with a short or long secondary condiophore, similar and smaller to the primary conidia. Secondary conidiophores branched at the base or tip, thus bearing 2 secondary conidia at each tip. Sometimes form 2–5 secondary conidia like “tomatoes on sticks” from small to large at each branch. Microconidia not observed on the PDA culture and on the 2% water agar. Zygospores formed between adjacent segments after 7 days, smooth, globose, 30–45 μm in diameter, with a 2–4 μm thick wall.

##### Notes.

*Conidiobolusmarcoconidius* is distinguished morphologically by its larger primary conidia compared to other *Conidiobolus* s.s. species, with the exception of *C.coronatus* (King 1977). Notably, it can be readily differentiated from *C.coronatus* by the absence of villose spores (Batko 1964). Additionally, *C.marcoconidius* is characterized by secondary conidiophores that branch at the base, giving rise to 2–5 secondary conidia resembling “tomatoes on sticks” at each branch, varying in size from small to large. In the phylogenetic tree, it forms a discrete clade, setting it apart from other *Conidiobolus* s.s. species.

#### ﻿ Conidiobolus polyspermus

Taxon classificationFungiEntomophthoralesConidiobolaceae

Drechsler, Mycologia, 53: 279. 1961.

01F48CE4-7DFE-5BAA-B274-83057F9E8A78

MycoBank No: 328763

##### Specimens examined.

United States, Maryland, 26 July 1955, Drechsler, ATCC 14444.

##### Description.

Refer to Drechsler (1961).

##### Notes.

In accordance with King’s numerical taxonomy of *Conidiobolus* (King 1976b), *C.polyspermus* was initially identified as a synonym of *C.megalotocus*. However, upon a thorough comparison of morphological traits based on the original descriptions, it became evident that *C.polyspermus* (15–55 × 12–48 μm) produces larger conidia than those of *C.megalotocus* (12–44 × 10–42 μm). Notably, *C.polyspermus* is not reported to form microconidia, distinguishing it from *C.megalotocus* (Drechsler 1956; 1961). Furthermore, the phylogenetic tree (Fig. 1) revealed a distinct relationship between *C.polyspermus* and *C.megalotocus*. In light of these findings, we propose the separation of *C.polyspermus* from *C.megalotocus*, affirming its taxonomic status at the species level.

## ﻿Discussion

Over an extended period, DNA-based techniques have played a pivotal role in uncovering both inter- and intra-species phylogenetic variations, essential for describing new species (Kidd et al. 2023). While the ITS region stands as a universal barcode marker for fungal identification, its applicability to entomophthoroid fungi is hindered by high intragenomic variation (Schoch et al. 2012; Hyde et al. 2023). Fortunately, the development of the full ribosomal operon and additional gene loci encoding proteins as fungal barcodes has addressed some of these challenges (James et al. 2006; Wurzbacher et al. 2019; Voigt et al. 2021; Zhao et al. 2023). In understanding the phylogeny of entomophthoroid fungi, reclassifications based on molecular sequences of nucLSU-SSU, mtSSU, and RPB2 have led to an updated taxonomic system proposed by Humber (2012), building upon the work of Gryganskyi et al. (2012, 2013). However, with only 31 fungal taxa having molecular data, constituting a mere fraction of entomophthoroid fungi, further phylogenetic analyses are imperative for a comprehensive understanding.

In light of the aforementioned phylogenetic framework, our study employed the same loci (excluding *EFL* instead of RPB2 for ease of amplification) to investigate the phylogeny of conidiobolus-like fungi. This endeavor resulted in the establishment of four new genera, i.e. *Azygosporus* B. Huang & Y. Nie, *Capillidium* B. Huang & Y. Nie, *Microconidiobolus* B. Huang & Y. Nie, and *Neoconidiobolus* B. Huang & Y. Nie, through a combination of molecular and morphological evidence. Additionally, *Conidiobolus* s.s. was proposed to accommodate members in the subgenus Delacroixia (Nie et al. 2020a; Cai et al. 2021). The evolution of phylogenomic studies in various fungal groups (Spatafora et al. 2016; Vandepol et al. 2020; Li et al. 2021) has further revealed a polyphyletic relationship among conidiobolus-like fungi, leading to the introduction of three new families, i.e. *Capillidiaceae* Y. Nie, Stajich & K.T. Hodge, *Conidiobolaceae* B. Huang, Stajich & K.T. Hodge, and *Neoconidiobolaceae* X.Y. Liu, Stajich & K.T. Hodge, grounded in morphological evidence and ancestral lifestyle considerations (Gryganskyi et al. 2022).

Furthermore, our molecular analyses underscored the high sensitivity of both nucLSU and *EFL* sequences in delineating conidiobolus-like fungi (Nie et al. 2012). Considering the balance between amplification efficiency, data integrity, and diversity, three loci of nucLSU, mtSSU, and *EFL* were selected for species recognition within *Conidiobolus* s.s. (Nie et al. 2020b, 2023).

In this study, we recovered the species status of *C.polyspermus*, while *C.eurypus* was synonymized with *C.megalotocus*. This synonymy will be subject to re-evaluation with the inclusion of molecular data for *C.eurypus*. Notably, *C.polyspermus* was also not reported to produce microconidia, a trait shared with six other *Conidiobolus* s.s. species. With the addition of descriptions for two new species in this manuscript, the count of *Conidiobolus* s.s. species lacking observation of microconidia has risen to nine. The morphological variation or genetic mutation behind this phenomenon remains a question that could be addressed not only through phylogenomic analyses but also by conducting comparative genomics analyses within a broader spectrum of *Conidiobolus* s.s. species.

With the introduction of two new *Conidiobolus* s.s. species, namely *C.jiangxiensis* and *C.marcoconidius* in the family *Conidiobolaceae* herein, the number of known *Conidiobolus* s.s. species are up to 22. However, limited reports of new species within the genera *Azygosporus* and *Microconidiobolus* in China underscore the need for an in-depth exploration of advanced species diversity within *Conidiobolaceae* from China in our future studies.

## Supplementary Material

XML Treatment for Conidiobolus jiangxiensis

XML Treatment for Conidiobolus marcoconidius

XML Treatment for Conidiobolus polyspermus

## ﻿Additional information

### Author ORCIDs

Yong Nie https://orcid.org/0000-0001-8964-1661

Heng Zhao https://orcid.org/0000-0003-2938-5613

Bo Huang https://orcid.org/0000-0001-6032-7396

### Data availability

All of the data that support the findings of this study are available in the main text.
